# Comments on Prevalence of Non-alcoholic Fatty Liver Disease and Its Related Factors in Iran

**Published:** 2017-02-01

**Authors:** H. Karimi-Sari, S.M. Mousavi-Naeini, A. Khonche

**Affiliations:** 1 *Student Research Committee, Baqiyatallah University of Medical Sciences, Tehran, Iran*; 2 *Baqiyatallah Research Center for Gastroenterology and Liver Diseases (BRCGL), Baqiyatallah University of Medical Sciences, Tehran, Iran*; 3 *Trauma Research Center, Baqiyatallah University of Medical Sciences, Tehran, Iran*

**DEAR EDITOR**

We read with great interest a recently published meta-analysis in the *IJOTM*. Moghaddasifar, *et. al.*, estimated the prevalence of non-alcoholic fatty liver disease (NAFLD) and related factors in Iranian population as 33.95% [1]. The prevalence of NAFLD ranges from 2.04% to 82.9% in different studies.

They correctly identified different study populations as the main source of this heterogeneity and tried to correct it by using meta-regression analysis and coding the different populations. Our study, one of the included studies in the meta-analysis, seems not suitable to pooling with other included studies [2]. We evaluated 114 morbidly obese patients, candidates for sleeve surgery—a type of bariatric surgery. Patients with good physical conditions are usually selected for this specific procedure to decrease the rate of complications. Therefore, our study is not a good sample for adults, especially those with morbid obesity, and may underestimate the prevalence of NAFLD in this population.

Another issue is the main calculations that seem to have an error due to ignoring the size of populations in different provinces. For better estimation of the prevalence of NAFLD in Iran, authors should have considered the size of provinces and age-gender distribution in each region [3, 4].

We also think the authors might have “double-publication” bias. The 952 children and adolescents out of 1107 subjects of Tazhibi, *et. al.*, study seems to be the same subjects of Adibi, *et. al.*, study [5, 6]. One study compared the prevalence of NAFLD in obese and non-obese children [5], while another evaluated the effect of life-style on NAFLD in these children [6]—both studies reported a prevalence of 16.9%.

For lack of enough data homogeneity about the prevalence of NAFLD in Iran, this topic seems not suitable for a meta-analysis study. Good quality original studies evaluating prevalence of NAFLD and correlated factors in Iran are strongly needed. Large cohort studies may resolve this important issue.

## Conflicts of Interest:

None declared.

## FINANCIAL SUPPORT:

None.
