# Supplementary material for: Comments on Prevalence of Non-alcoholic Fatty Liver Disease and Its Related Factors in Iran
Source: Int J Organ Transplant Med. 2017 Feb 1;8(1):52–3. (PMC5347407)
Supplement: Related texts

# Authors’ Reply

**Published:** 2017-02-01

**Authors:** M. Moosazadeh

**Affiliations:** *Assistant Professor, Health Sciences Research Center, Faculty of Health, Mazandaran University of Medical Sciences, Sari, Iran*

**DEAR EDITOR**

We deeply appreciate the comments on our paper [1]. Regarding the paper of Karimi-Sari, *et. al.* [2], which was included into our systematic review, the study population, obese patients undergoing sleeve gastrectomy, has been considered the adult population group. As the matter of fact, it was not ignored by the authors; they emphasized that the structure of the primary studies suffered from a great heterogeneity and mentioned this fact in detail within the text and Tables. Moreover, results of meta-regression models revealed the population as one of the main sources of the heterogeneity. In addition, the study population was one of the issues that was considered for subgroup analysis, though due to the limited number of primary studies in each subpopulation, only systematic review was carried out for such subgroup. 

We agree with other comments on entering two studies with different titles conducted in the same population [3, 4]. Although we considered the site the study was conducted, different sample sizes reported for each study, *i.e.*, the sample size of the study of Tazhibi, *et. al.* [4], was reported to be 1107, while the sample size of Adibi, *et. al. *[3], study was 952. That was why the researchers extracting the information made such a mistake. In total, the available evidence of primary studies is very low and such limitation is expected to be settled by future studies.

## References

[1] Moghaddasifar I, Lankarani KB, Moosazadeh M (2016). Prevalence of non-alcoholic fatty liver disease and its related factors in Iran. Int J Organ Transplant Med.

[2] Karimi-Sari H, Mousavi-Naeini SM, Ramezani-Binabaj M (2015). Prevalence of non-alcoholic fatty liver disease in morbidly obese patients undergoing sleeve bariatric surgery in Iran and association with other comorbid conditions. Jundishapur Journal of Chronic Disease Care.

[3] Adibi A, Kelishadi R, Beihaghi A (2009). Sonographic fatty liver in overweight and obese children, a cross sectional study in Isfahan. Endokrynologia Polska.

[4] Tazhibi M, Kelishadi R, Khalili Tahmasebi H (2010). Association of lifestyle with metabolic syndrome and non-alcoholic fatty liver in children and adolescence. Bimonthly Journal of Hormozgan University of Medical Sciences.
